# The evolving roles of imaging and fluid biomarkers for Alzheimer disease over the past quarter century

**DOI:** 10.1002/alz70856_101336

**Published:** 2025-12-25

**Authors:** Laura Ibanez

**Affiliations:** ^1^ Washington University School of Medicine, St. Louis, MO, USA

## Abstract

**Background:**

At the beginning of the century, Alzheimer disease (AD) biomarkers were only used in research studies and included hippocampal volume and cerebrospinal fluid (CSF) concentrations of Aβ42, total tau, and tau phosphorylated at position 181 (*p*‐tau181). Twenty‐five years later, imaging and fluid biomarkers have become critical tools in AD clinical trials and are increasingly being used in clinical care.

**Methods:**

The development of imaging and fluid AD biomarkers will be reviewed. The evolving roles of different modalities of biomarkers in research, clinical trials, and clinical care will be described.

**Results:**

In 2004, the first radiotracer that bound amyloid plaques was reported, which enabled visualization of the amount and regional distribution of amyloid plaques in the brains of living individuals via positron emission tomography (PET). Radiotracers binding to insoluble tau aggregates were described in 2013 and 2014. The use of amyloid and tau PET as a reference standard, as well as improvements in fluid biomarker assay technology, led to the first of many accurate AD blood tests in 2017 and 2018. These imaging and fluid biomarkers of AD have undergone waves of development, validation, and regulatory approval. AD research studies now use biomarkers extensively to study the biology of disease. Clinical trials use biomarkers to confirm that participants have AD pathology and to monitor the effects of treatment. AD biomarkers are increasingly being used in the clinical diagnosis of AD. The high acceptability and accessibility of AD blood tests may enable AD biomarker testing to become the standard of care in patients with cognitive impairment. In the future, if trials of preventative treatments are positive, AD biomarker testing of cognitively unimpaired older individuals may become routine.

**Conclusions:**

Advances in imaging and fluid biomarkers over the past quarter century have enabled greater understanding of AD biology and led to the successful development, approval, and clinical use of disease‐modifying AD treatments that target amyloid pathology. The clinical availability of AD‐specific treatments and high‐accuracy AD blood tests is currently transforming the clinical diagnosis and care of patients with AD.

